# Genome Sequencing of *Sulfolobus* sp. A20 from Costa Rica and Comparative Analyses of the Putative Pathways of Carbon, Nitrogen, and Sulfur Metabolism in Various *Sulfolobus* Strains

**DOI:** 10.3389/fmicb.2016.01902

**Published:** 2016-11-30

**Authors:** Xin Dai, Haina Wang, Zhenfeng Zhang, Kuan Li, Xiaoling Zhang, Marielos Mora-López, Chengying Jiang, Chang Liu, Li Wang, Yaxin Zhu, Walter Hernández-Ascencio, Zhiyang Dong, Li Huang

**Affiliations:** ^1^State Key Laboratory of Microbial Resources, Institute of Microbiology, Chinese Academy of SciencesBeijing, China; ^2^College of Life Sciences, University of Chinese Academy of SciencesBeijing, China; ^3^State Key Laboratory of Mycology, Institute of Microbiology, Chinese Academy of SciencesBeijing, China; ^4^Center for Research in Cell and Molecular Biology, Universidad de Costa RicaSan José, Costa Rica

**Keywords:** *Sulfolobus*, strain A20, genome sequencing, comparative genomics, carbon metabolism, nitrogen metabolism, sulfur metabolism

## Abstract

The genome of *Sulfolobus* sp. A20 isolated from a hot spring in Costa Rica was sequenced. This circular genome of the strain is 2,688,317 bp in size and 34.8% in G+C content, and contains 2591 open reading frames (ORFs). Strain A20 shares ~95.6% identity at the 16S rRNA gene sequence level and <30% DNA-DNA hybridization (DDH) values with the most closely related known *Sulfolobus* species (i.e., *Sulfolobus islandicus* and *Sulfolobus solfataricus*), suggesting that it represents a novel *Sulfolobus* species. Comparison of the genome of strain A20 with those of the type strains of *S. solfataricus, Sulfolobus acidocaldarius, S. islandicus*, and *Sulfolobus tokodaii*, which were isolated from geographically separated areas, identified 1801 genes conserved among all *Sulfolobus* species analyzed (core genes). Comparative genome analyses show that central carbon metabolism in *Sulfolobus* is highly conserved, and enzymes involved in the Entner-Doudoroff pathway, the tricarboxylic acid cycle and the CO_2_ fixation pathways are predominantly encoded by the core genes. All *Sulfolobus* species encode genes required for the conversion of ammonium into glutamate/glutamine. Some *Sulfolobus* strains have gained the ability to utilize additional nitrogen source such as nitrate (i.e., *S. islandicus* strain REY15A, LAL14/1, M14.25, and M16.27) or urea (i.e., *S. islandicus* HEV10/4, *S. tokodaii* strain7, and *S. metallicus* DSM 6482). The strategies for sulfur metabolism are most diverse and least understood. *S. tokodaii* encodes sulfur oxygenase/reductase (SOR), whereas both *S. islandicus* and *S. solfataricus* contain genes for sulfur reductase (SRE). However, neither SOR nor SRE genes exist in the genome of strain A20, raising the possibility that an unknown pathway for the utilization of elemental sulfur may be present in the strain. The ability of *Sulfolobus* to utilize nitrate or sulfur is encoded by a gene cluster flanked by IS elements or their remnants. These clusters appear to have become fixed at a specific genomic site in some strains and lost in other strains during the course of evolution. The versatility in nitrogen and sulfur metabolism may represent adaptation of *Sulfolobus* to thriving in different habitats.

## Introduction

Archaea of genus *Sulfolobus* are widespread in solfataric fields around the globe. Known *Sulfolobus* species were mostly isolated from the Northern hemisphere (Brock et al., 1972; Grogan et al., 1990; Huber and Stetter, 1991; Jan et al., 1999; Suzuki et al., 2002; Xiang et al., 2003; Guo et al., 2011; Mao and Grogan, 2012; Zuo et al., 2015). These *Sulfolobus* isolates have been classified into nine species. Since *Sulfolobus* is readily grown and manipulated under laboratory conditions (Grogan, 1989), it has been used as a model for the study of Archaea. *Sulfolobus* also serves as a model for the study of eukaryotic genetic mechanisms because of the striking resemblance between Archaea and Eukarya in the flow of genetic information (Bell et al., 2002). In addition, *Sulfolobus* has been used as a host for the study of an increasing number of archaeal viruses and plasmids (Arnold et al., 2000; Rice et al., 2001; Xiang et al., 2003; Guo et al., 2011; Wang et al., 2015).

The complete genomes of 17 *Sulfolobus* strains belonging to four species have so far been deposited in GenBank. These include a *Sulfolobus tokodaii* strain (str.7) (Kawarabayasi et al., 2001), three *Sulfolobus solfataricus* strains (She et al., 2001; McCarthy et al., 2015), four *Sulfolobus acidocaldarius* strains (Chen et al., 2005; Mao and Grogan, 2012), and nine *Sulfolobus islandicus* strains (Reno et al., 2009; Guo et al., 2011; Zhang et al., 2013). Genomic comparisons show that *Sulfolobus* species are genetically diverged in relation to their geographic distance (Whitaker et al., 2003; Reno et al., 2009). Discontinuous and distantly separated habitats seem to be geographic barriers limiting gene flow among *Sulfolobus* populations. The variation in gene content among geographically diverse isolates is consistent with an isolation-by-distance model of diversification (Whitaker et al., 2003; Grogan et al., 2008; Reno et al., 2009). Apparently, genomic analyses of more geographically separated isolates would help shed more light on the genetic diversity and phylogenetic relationships of *Sulfolobus* strains.

All species of *Sulfolobus* are aerobic sulfur oxidizers, and many of them are initially described as autotrophs or mixotrophs (Brock et al., 1972). Two autotrophic carbon fixation cycles have been described in Crenarchaeota, i.e., the 3-hydroxypropionate/4-hydroxybutyrate (HP/HB) cycle and the dicarboxylate/4-hydroxybutyrate (DC/HB) cycle (Berg et al., 2007, 2010; Huber et al., 2008; Ramos-Vera et al., 2011). The HP/HB cycle was confirmed by biochemical assays in Sulfolobales including *Sulfolobus, Acidianus*, and *Metallosphaera* (Berg et al., 2007; Teufel et al., 2009; Estelmann et al., 2011; Demmer et al., 2013). H_2_, hydrogen sulfide, sulfur, tetrathionate, and pyrite have been described as electron donors for autotrophically-grown *Sulfolobus* (Brock et al., 1972; Wood et al., 1987; Huber and Stetter, 1991; Huber et al., 1992). For the heterotrophical growth of *Sulfolobus*, the conversion of glucose to pyruvate was thought to rely on a non-phosphorylative Entner-Doudoroff (ED) pathway, as shown in *S. solfataricus* and *S. acidocaldarius* (Siebers et al., 1997). However, extensive *in vivo* and *in vitro* assays later indicated that both the semi-phosphorylative and the non-phosphorylative ED pathways might operate in *S. solfataricus* (Ahmed et al., 2005; Ettema et al., 2008). Genomic analyses of the metabolic pathways have been reported for several *Sulfolobus* strains (Sensen et al., 1998; Kawarabayasi et al., 2001; She et al., 2001; Chen et al., 2005; Guo et al., 2011; Jaubert et al., 2013). A further genomic comparison of metabolic pathways in various *Sulfolobus* strains will be of significance to the understanding of the strategies of the organisms to adapt to thriving in their environments. In the present study, we isolated a novel *Sulfolobus* species, denoted strain *Sulfolobus* sp. A20, from an acidic hot spring in Laguna Fumarólica, Costa Rica, and sequenced the genome of the strain. The 16S rRNA gene of strain A20 exhibits the highest sequence identity (~95.6%) to those of *S. islandicus* and *S. solfataricus* isolates, but the significant differences suggest that strain A20 represents an independent *Sulfolobus* species. The genome of strain A20 was compared with all other available *Sulfolobus* genomes, and analyses of the pathways of carbon, nitrogen and sulfur metabolism in various *Sulfolobus* strains were performed.

## Materials and methods

### Isolation of strain A20

A water sample FL1010-1 was collected in October 2010 from a hot spring, known as Laguna Fumarólica (10°46,365′ N and 85°20,646′ W, ~85°C, pH 3–4), in the Las Palias hydrothermal field (Las Pailas sector), which is located in the southwest flank of the Rincón de la Vieja volcano crater. Rincón de la Vieja volcano (10°49′ N, 85°19′ W), an andesitic volcano in northwestern Costa Rica, belongs to the Circum Pacific Ring of Fire, which is a geothermal belt different from its nearest neighbors, the Yellowstone National Park and the Lassen Volcanic National Park. The sample was concentrated by tangential flow ultrafiltration through a hollow fiber membrane with a molecular mass cutoff of 6 kDa (Tianjin MOTIMO Membrane Technology, China). An enrichment culture was established by inoculating the concentrate in Zillig's medium (Zillig et al., 1994), which contained 0.3% (NH_4_)_2_SO_4_, 0.05% KH_2_PO_4_·3H_2_O, 0.05% MgSO_4_·7H_2_O, 0.01% KCl, 0.001% Ca(NO_3_)_2_·4H_2_O, 0.07% Glycine, 0.05% yeast extract, 0.2% sucrose, and 0.2% of a trace element solution (0.09% MnCl_2_·4H_2_O, 0.225% Na_2_B_4_O_7_·10H_2_O, 0.011% ZnSO_4_·7H_2_O, 0.0025% CuCl_2_·2H_2_O, 0.0015% NaMoO_4_·2H_2_O, 0.0005% CoSO_4_·7H_2_O). After incubation for 7–10 days at 75°C with shaking at 150 rpm, samples of the grown culture were spread on Zillig's medium plates solidified with 0.8% gelrite. The plates were incubated for 7 days at 75°C. Colonies were picked and purified by re-plating. Observation of the cells of strain A20 was carried out under a transmission electron microscope (JEM-1400, Jeol Ltd., Tokyo, Japan) at 80 kV by negatively staining with 2% uranyl acetate.

### Genome sequencing and annotation

The genomic DNA of strain A20 was isolated and purified, as described (Chong, 2001), and sequenced on the Pacific Biosciences (PacBio) RS II and Illumina Hiseq 2000 systems at AnnoGenne, Beijing, China. The genome was assembled with SMRT analysis v2.3.0 and RS_HGAP_Assembly.3, and the genome assembly was improved by using the software Pilon (Walker et al., 2014). Identification of protein-coding open reading frames (ORFs) and annotation of the ORFs were performed by NCBI using the NCBI Prokaryotic Genome Annotation Pipeline (https://www.ncbi.nlm.nih.gov/genome/annotation_prok/). Genes were functionally annotated by BLAST search in COG, KEGG, Nr, and Pfam Databases (Camacho et al., 2009; Finn et al., 2011). Putative insertion sequence (IS) elements were identified by BLASTn search against the IS finder Database (http://www-is.biotoul.fr).

### Comparative genomics analysis

The nucleotide sequences of all genome-sequenced *Sulfolobus* strains and the corresponding amino acid sequences were retrieved from the GenBank database and the NCBI Reference Sequence database (RefSeq) (Table 1). The dot plots of any two genomes for their genomic synteny were profiled with Mummer (Kurtz et al., 2004), and DNA-DNA hybridization (DDH) values *in silico* were computed using the Genome-to-Genome Distance Calculator (GGDC) version 2.0 (Meier-Kolthoff et al., 2013) by submitting the genome sequences to DSMZ (http://ggdc.dsmz.de) (Auch et al., 2010). All protein sequences derived from the *Sulfolobus* genomes were compared using all-by-all BLASTp with a threshold *E*-value 10^−10^, and grouped into orthologous gene families by OrthoMCL (Li et al., 2003). Gene groups consisting of orthologous genes present in all genomes, in more than two but not all genomes or in only one genome were defined as core, variable, or individual gene groups, respectively. A Venn diagram of the orthologous analysis of gene families was built with R version 3.0.2.

**Table 1:** *****Sulfolobus*** strains with complete genome sequences**.

**Strains**	**GenBank accession no**.	**NCBI RefSeq no**.	**Genome size (Mb)**	**No. of ORFs**	**No. of rRNAs**	**No. of tRNAs**	**GC%**	**Habitat**
*Sulfolobus* sp. A20	CP017006	NZ_CP017006	2.69	2591	3	45	34.8	Las Palias, Costa Rica
*S. solfataricus* P2	AE006641	NC_002754	2.99	2896	3	45	35.8	Naples, Italy
*S. solfataricus* P1	LT549890	NZ_LT549890	3.03	2967	3	45	35.8	Naples, Italy
*S. solfataricus* 98/2	CP001800	NC_017274	2.67	2605	3	45	35.8	Yellowstone, USA
*S. islandicus* REY15A	CP002425	NC_017276	2.52	2535	3	46	35.3	Reykjanes, Iceland
*S. islandicus* HVE10/4	CP002426	NC_017275	2.66	2692	3	44	35.1	Hvergaardi, Iceland
*S. islandicus* LAL14/1	CP003928	NC_021058	2.47	2505	3	45	35.1	Iceland
*S. islandicus* L.S.2.15	CP001399	NC_012589	2.74	2767	3	45	35.1	Lassen, USA
*S. islandicus* 14.25	CP001400	NC_012588	2.61	2682	3	45	35.1	Kamchatka, Russia
*S. islandicus* M16.4	CP001402	NC_012726	2.59	2678	3	45	35.0	Kamchatka, Russia
*S. islandicus* M16.27	CP001401	NC_012632	2.69	2766	3	45	35.0	Kamchatka, Russia
*S. islandicus* Y57.14	CP001403	NC_012622	2.7	2708	3	48	35.4	Yellowstone, USA
*S. islandicus* YN15.51	CP001404	NC_012623	2.81	2791	3	46	35.3	Yellowstone, USA
*S. acidocaldarius* DSM639	CP000077	NC_007181	2.23	2224	3	48	36.7	Yellowstone, USA
*S. acidocaldarius* N8	CP002817	NC_020246	2.18	2188	3	48	36.7	Hokkaido, Japan
*S. acidocaldarius* Ron121	CP002818	NC_020247	2.22	2227	3	30	36.7	Ronneburg, Germany
*S. acidocaldarius* SUSAZ	CP006977	NC_023069	2.06	2038	3	46	36.3	Los Azufres, Mexico
*S. tokodaii* str.7	BA000023	NC_003106	2.69	2764	3	46	32.8	Kyushu, Japan

### Phylogenetic analysis

The 16S rRNA gene sequences of *Sulfolobus* species were extracted from the genome sequences and aligned using the CLUSTAL X program (Thompson et al., 1997). Phylogenetic trees were constructed using the neighbor-joining, maximum-parsimony, and maximum-likelihood methods implemented in the software package MEGA version 5.0 (Tamura et al., 2011). Evolutionary distances were calculated using Kimura's two-parameter model. The resulting tree topologies were evaluated by bootstrap analysis with 1000 re-samplings.

### Metabolic pathway assignments

The Kyoto Encyclopedia of Genes and Genomes (KEGG) database (Ogata et al., 1999; Kanehisa and Goto, 2000) was used in the analysis of the metabolic pathways of *Sulfolobus* species. All amino acid sequences derived from the genomes of *Sulfolobus* were submitted to the KEGG database, and the metabolic functions of these sequences were annotated by kass (Moriya et al., 2007). The KO (KEGG Orthology) term and corresponding KEGG pathway for each ORF were automatically generated and provided.

### Sequencing data accession number

The genome data of *Sulfolobus* sp. A20 have been deposited in the Genbank database under accession number CP017006.

## Results

### General features of *Sulfolobus* sp. A20

**Figure 1:**
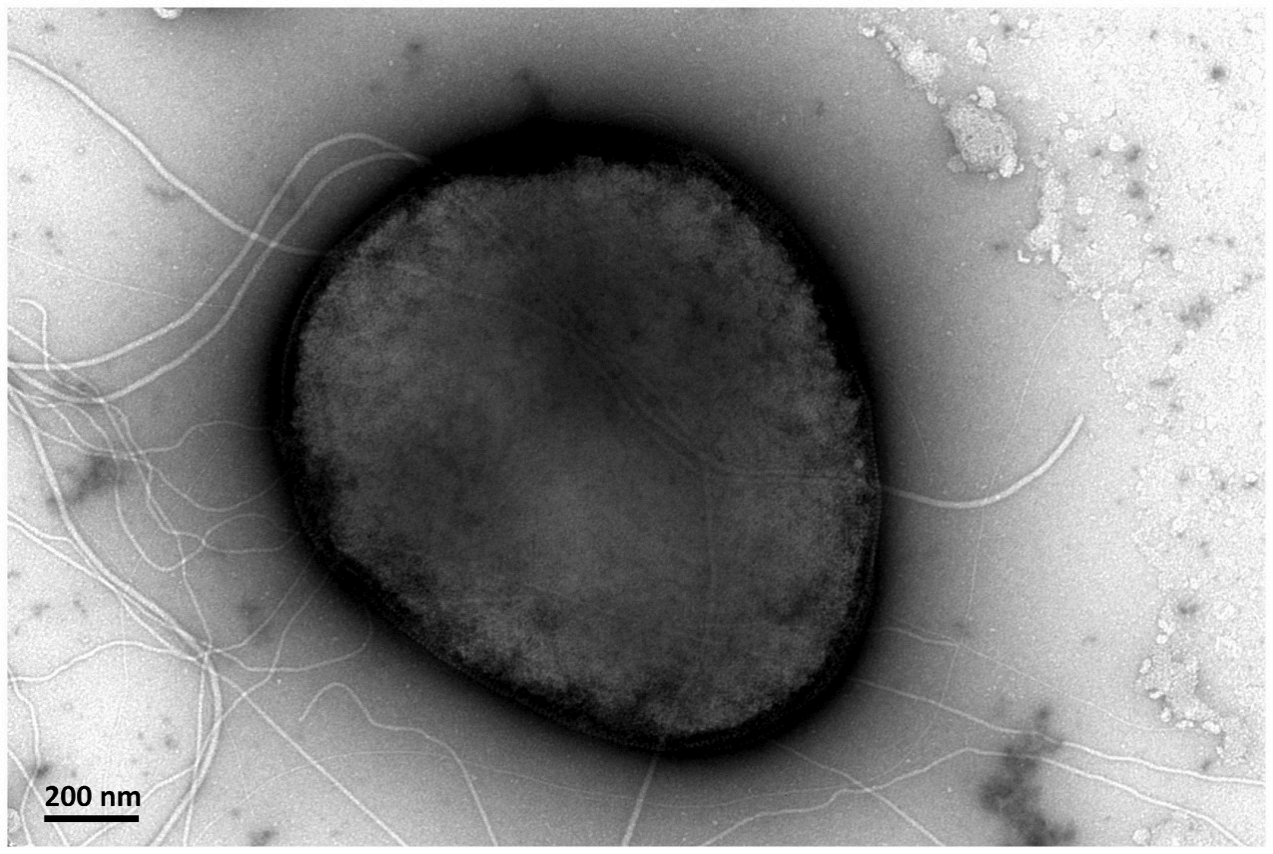
**A transmission electron micrograph showing the morphology of ***Sulfolobus*** sp. A20**.

*Sulfolobus* sp. A20 was isolated from a hot spring in Costa Rica. The cells of strain A20 were irregular cocci (0.8–1.0 μm in diameter) with flagella (Figure 1). Growth occurred at temperatures between 65 and 85°C, and pH between 2.0 and 4.5. The strain grew optimally at 75–85°C and pH 4.0. The doubling time of the strain was ~14.3 h under the optimal growth conditions.

### The genome of *Sulfolobus* sp. A20

The genome of strain A20 was sequenced using a combination of PacBio RS II and Illumina Hiseq 2000 sequencing technologies with a 2 × 100 bp mode at a 150-fold and a 700-fold coverage, respectively. The genome consists of a single circular chromosome of 2,688,317 bp with 2591 ORFs, a single 16-23S rRNA cluster, a 5S rRNA gene, 45 tRNA genes and 5 miscellaneous RNA genes (misc RNAs). The average size of an ORF is ~291 amino acids. No extra-chromosomal genetic elements were detected in the strain. The G+C content of the genome is 34.78%. BLASTp searches identified matches in the protein database at GenBank for ~97.22% of the total ORFs of strain A20 (2519 ORFs). Among these ORFs, 2223 (~85.80% of total ORFs) are most closely related to those from the genus *Sulfolobus*, and 227 are closely related to those from other genera of the Sulfolobales. The general features of the strain A20 genome are compared with those of the other sequenced Sulfolobus genomes in Table 1.

Strain A20 encodes a complete set of enzymes and proteins involved in DNA transactions, including DNA replication, DNA repair and recombination, and RNA transcription. These proteins are highly conserved among the *Sulfolobus* strains, whose genomes have been sequenced, and share the highest sequence identity with those from *S. islandicaus* or *S. solfataricus*. For example, DNA replication proteins, including ORC1-type DNA replication proteins (BFU36_RS04705, BFU36_RS02195, and BFU36_RS09865), mini-chromosome maintenance protein (MCM, BFU36_RS02210), primase subunits (BFU36_RS01270, BFU36_RS03220, and BFU36_RS03380), proliferating cell nuclear antigen subunits (PCNA, BFU36_RS01275, BFU36_RS03780, and BFU36_RS03820), replication factor C (RFC, BFU36_RS02175, and BFU36_RS02180) and DNA polymerases (BFU36_RS05445, BFU36_RS13105, and BFU36_RS03245), from strain A20 closely resemble their homologs at the amino acid sequence level from the other *Sulfolobus* strains. Strain A20 also encodes small, basic and nucleic acid-binding proteins, i.e., Cren7 (BFU36_RS01545), two Sul7d proteins (BFU36_RS09545 and BFU36_RS11200), and two members of the Sac10b family (BFU36_RS01605 and BFU36_RS01615).

Like other *Sulfolobus* strains, strain A20 carries integrative elements, CRISPR-based immune systems and antitoxin/toxin systems (Guo et al., 2011). About 13 ORFs are annotated as the homologs of transposase, and nine copies of putative insertion sequence (IS) elements are found. Among these IS elements, eight belong to the IS200/605 family and one to the IS607 family. Six CRISPR loci of the two subtypes (I-A and III-B) and cmr1-6 proteins are identified (Grissa et al., 2007). No apparent sequence homology was detected between the spacers and the known sequences of *Sulfolobus*/*Acidianus* viruses. Five copies of family II (VapBC) antitoxin-toxin gene pairs are found in the strain A20 genome.

Dot plot analysis reveals no genomic synteny between strain A20 and any of the genome-sequenced *Sulfolobus* strains. Pairwise DNA-DNA hybridization (DDH) *in silico* between strain A20 and one of the tested *Sulfolobus* strains, including *S. tokodaii* str.7, *S. acidocaldarius* DSM 639, three *S. solfataricus* strains, and four *S. islandicus* strains, produces DDH values between 16.7 and 23.1% (Table 2), which are far below the 70% threshold proposed for species definition (Tindall et al., 2009). These results suggest that strain A20 represents a novel *Sulfolobus* species.

**Table 2:** *****In silico*** DNA-DNA hybridization (DDH) values (%) between ***Sulfolobus*** strains^a^**.

**Sample**	**Strain**	**SSO**			**SIS**				**SAC**	**STO**
	**A20**	**P1**	**P2**	**98/2**	**LS2.15**	**REY15A**	**HVE10/4**	**LAL14/1**	**DSM639**	**str7**
Strain A20	–	16.80	16.80	16.80	16.70	16.70	16.70	16.70	23.10	19.60
SSO-P1		–	94.80	91.50	38.00	37.50	37.30	37.40	18.80	24.00
SSO-P2			–	91.10	40.00	39.30	38.90	39.20	18.20	23.00
SSO-98/2				–	37.20	37.00	36.90	36.90	18.90	24.30
SIS-LS2.15					–	85.80	87.80	81.70	18.40	20.00
SIS-REY15A						–	94.90	94.10	18.10	21.10
SIS-HVE10/4							–	94.00	18.20	21.70
SIS-LAL14/1								–	18.10	21.20
SAC-DSM639									–	15.70
STO-str7										–

a *SSO, S. solfataricus; SAC, S. acidocaldarius; SIS, S. islandicus; STO, S. tokodaii*.

### Phylogenetic analysis of *Sulfolobus* strains

**Figure 2:**
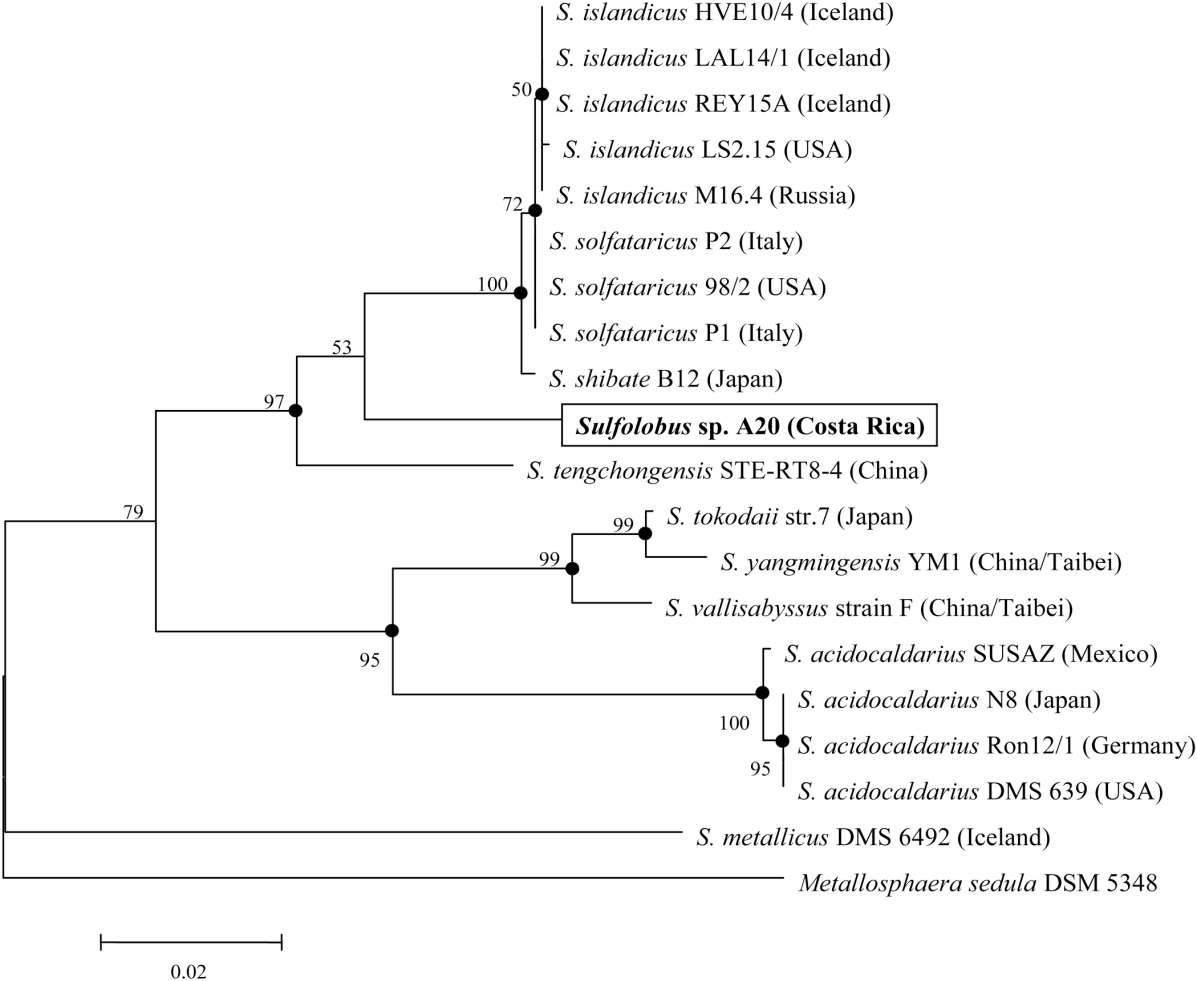
**Phylogenetic tree of genome-sequenced ***Sulfolobus*** strains based on the 16S rRNA gene sequences**. *Metallosphaera sedula* DSM 5348 is used as the outgroup. Numbers denote the bootstrap percentages obtained with 1000 replicates.

The 16S rRNA gene sequence of strain A20 was retrieved from the genome sequence of the strain. BLAST searches show that it is most similar (~95.6% identity) to those from several isolates of *S. islandicus* and *S. solfataricus*. The known *Sulfolobus* species appear to group into two main clades, as indicated by the phylogenetic analysis based on the 16S rRNA gene sequences (Figure 2). Strain A20, together with *S. islandicus, S. solfataricus, S. shibatae*, and *S. tengchongensis*, comprise one clade, while *S. acidocaldarius, S. tokodaii, S. vallisabyssus*, and *S. yangmingensis* make up the other. *S. metallicus* DSM6482, a strictly chemolithoautotrophic and ore-leaching *Sulfolobus* species, appears to be phylogenetically distant from the two main clades.

### Core, variable, and individual genes

**Figure 3:**
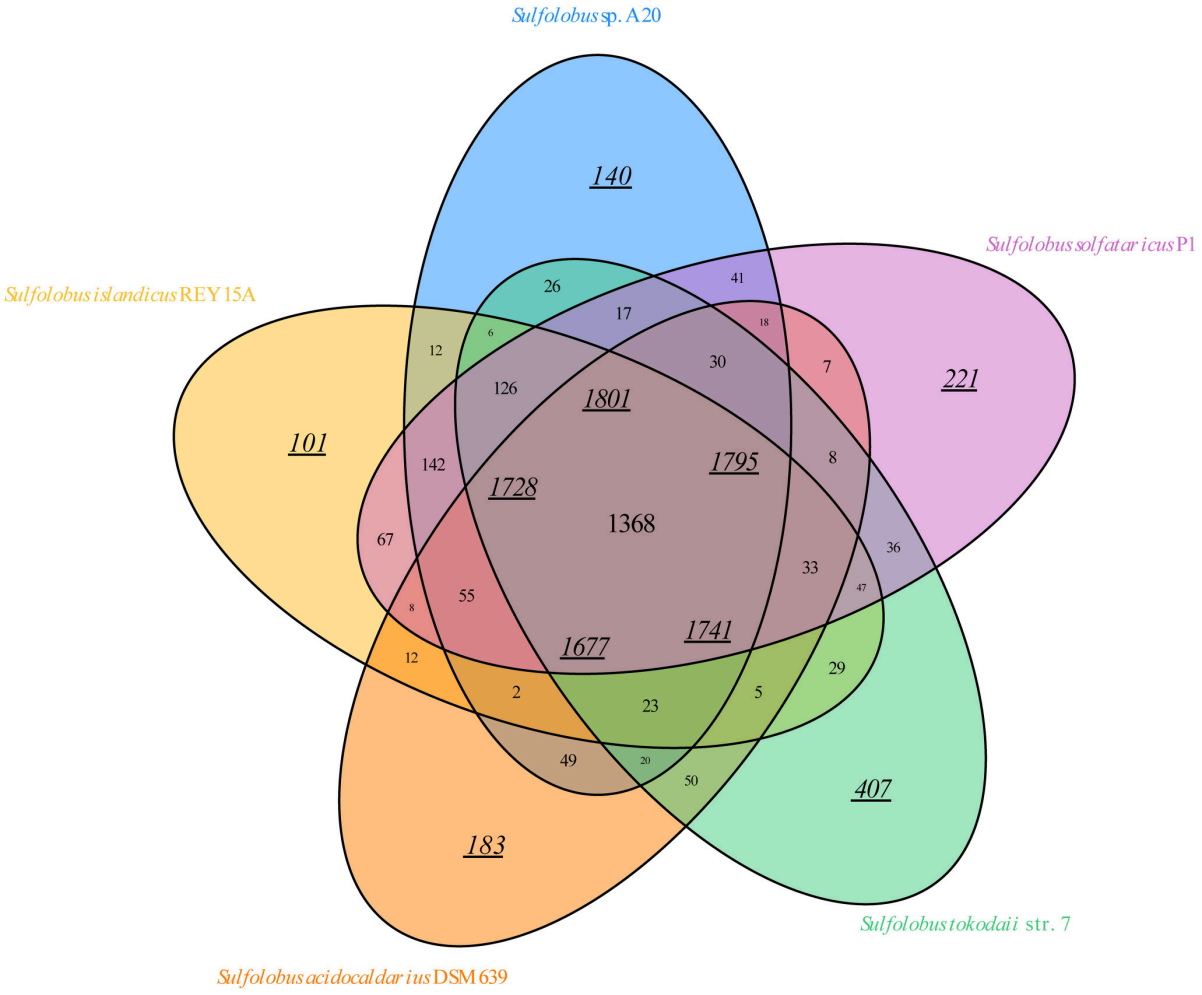
**Venn diagram of the conservation of protein-coding ORFs of the genome-sequenced type strains of ***Sulfolobus*** species**. The overlaps between the ellipses show the gene groups shared by different strains with the number of shared gene groups indicated. The number of genes for each strain in a section of the diagram is shown by an underlined number in italics. Each underlined number in italics in the middle of the diagram indicates the number of core genes for a strain analyzed.

A total of 18 *Sulfolobus* genomes, including the strain A20 genome, have been completely sequenced so far. To gain insight into the similarities and differences of the genomes from various *Sulfolobus* species, we compared the genome sequences available for the type strains of four *Sulfolobus* species, i.e., *S. acidocaldarius* DSM 639, *S. islandicus* REY15A, *S. solfataricus* P1, and *S. tokodaii* str.7 as well as strain A20. The numbers of predicted ORFs for the five genomes are 2663 ± 439. The ORFs from these genomes are grouped into homologous groups. A total of 1368 gene groups form the core gene groups of the genus *Sulfolobus* (Figure 3). This number corresponds to 1801 genes (~69.51% of the total genes) in strain A20 (Table S1). Notably, the difference between these two numbers (i.e., 1368 gene groups vs. 1801 genes) is greater in strain A20 than in other *Sulfolobus* strains analyzed in this study, suggesting greater gene redundancy in A20 than in the other strains. Eight hundred and sixty nine gene groups are found in more than one, but not all, of the five genomes. These groups may constitute the variable parts of the *Sulfolobus* genomes. Strain A20 shares most gene groups with *S. solfataricus* P1 (1797), in agreement with their closest phylogenetic relationship. Moreover, the tested *Sulfolobus* genomes contain variable numbers of individual gene groups. In strain A20, 140 genes (~5.40% of the total ORFs) are not found in other four *Sulfolobus* strains. By comparison, *S. tokodaii* str.7 has the most individual genes (407, or ~14.72% of the total ORFs), whereas *S. islandicus* REY15A has the fewest individual genes (101, or ~3.98% of the total ORFs). Notably, the majority (>80%) of the individual genes encode hypothetical proteins. Conceivably, the exact numbers of core, variable and individual genes in *Sulfolobus* strains will change as the sample size increases but the general pattern of the distribution of these three groups of genes will likely remain.

### Metabolic pathways

KEGG analyses reveal that the genome of strain A20 contains 84, 3 and 10 genes encoding functions in central carbon metabolism, nitrogen metabolism and sulfur metabolism, respectively. As compared to other known *Sulfolobus* genomes, the A20 genome appears to have similar numbers of the genes encoding proteins or protein subunits involved in carbon and sulfur metabolism but fewer genes for nitrogen metabolism. In addition, a total of 15 different ATP-binding cassette (ABC) transporters are identified in the strain A20 genome. By comparison, the numbers of ABC transporters are 10–14 in various *S. islandicus* strains (Guo et al., 2011), 11 in *S. solfataricus* P2 (She et al., 2001), 6 in *S. tokodaii* str.7 (Kawarabayasi et al., 2001), and 3 in *S. acidocaldarius* DSM639 (Chen et al., 2005). The ABC transporters in strain A20 include those for the transportation of trehalose (BFU36_RS00560–BFU36_RS00575, 4 ORFs in all), arabinogalactan oligomer/maltooligosaccharide (BFU36_RS00855–BFU36_RS00870, 4 ORFs), and glucose/arabinose (BFU36_RS07440–BFU36_RS07455, 4 ORFs, and BFU36_RS08120–BFU36_RS08130, 3 ORFs), suggesting the potential ability of strain A20 to utilize a wide range of sugars. There are 16 ORFs belonging to eight glycoside hydrolase (GHs) families, supporting the possibility that strain A20 uses a number of disaccharides and polysaccharides, e.g., cellobiose, maltotriose, mannan, and starch, for growth. A gene (BFU36_RS09315) encoding a putative trehalose glycosyl-transferring synthase (TreT) exists in the genome of strain A20. TreT from *Thermoproteus tenax* has been shown to catalyze trehalose synthesis from NDP-glucose or glucose (Kouril et al., 2008). Therefore, it is possible that strain A20 is capable of trehalose synthesis. There is also a cluster of four putative carotenoid biosynthetic genes (BFU36_RS07010–BFU36_RS07025), encoding homologs of lycopene cyclase, phytoene synthase, beta-carotene hydroxylase and phytoene desaturase, respectively, in the strain A20 genome, and these genes are arranged in the same manner as those in *S. solfataricus* (Hemmi et al., 2003) (Table S2).

#### Central carbon metabolism

As revealed by the genome analysis of *S. solfataricus* P2, strain A20 lacks the classical Embden–Meyerhof–Parnas (EMP) and pentose phosphate pathways, since the genes encoding the homologs of the key enzymes in these pathways, i.e., phosphofructokinase in the former and glucose-6-phosphate dehydrogenase, 6-phosphogluconolactonase and 6-phosphogluconate dehydrogenase in the latter, are missing from the genomes (She et al., 2001; Ulas et al., 2012). Like other genome-sequenced *Sulfolobus* strains, strain A20 may utilize glucose through either the semi-phosphorylative or the non-phosphorylative-Entner-Doudoroff (ED) pathway, or both (Table 3). Like all other *Sulfolobus* species, strain A20 contains all genes involved in the tricarboxylic acid (TCA) cycle, except for those encoding the alpha-ketoglutarate dehydrogenase complex. The genes for the alpha-ketoglutarate dehydrogenase complex are replaced by those encoding the two subunits of 2-oxoacid:ferredoxin oxidoreductase, an enzyme catalyzing coenzyme A-dependent oxidative decarboxylation of 2-oxoacids (Zillig, 1991; Nishizawa et al., 2005). Intriguingly, the copy number of the genes for 2-oxoacid:ferredoxin oxidoreductase varies among *Sulfolobus* species. A single copy of the genes are present in strain A20, *S. solfataricus* and *S. islandicus*, whereas two copies of the genes are found in *S. acidocaldarius* and *S. tokodaii*, in apparent agreement with the phylogenetic relationship among these species (Figure 2).

**Table 3:** **Enzymes involved in the Entner-Doudoroff pathway in strain A20**.

**KO term**	**ORF(BFU36_RS)**	**Enzyme**	**Pathway^a^**
K18125	06060	Glucose dehydrogenase	ED
K05308	06085	Gluconate dehydratase	ED
K18126	06095	2-keto-3-deoxygluconate kinase	sp ED
K11395	06090	2-keto-3-deoxy-6-phosphogluconate aldolase	sp ED
K18978	06100	Glyceraldehyde-3-phosphate dehydrogenase	sp ED
K15634	10260		ED
K15635	03725	Phosphglycorate mutase	
K01689	02015	Enoase	ED
K00873	01505	Pyruvate kinase	ED
K11395	06090	2-keto-3-deoxygluconate aldolase	np ED
K18020	09145		np ED
K18021	09155	Glyceraldehyde dehydrogenase	
K18022	09150		
K11529	02730	Glycerate kinase	np ED

a *sp, semi-phosphorylative pathway; np, non-phosphorylative pathway*.

**Figure 4:**
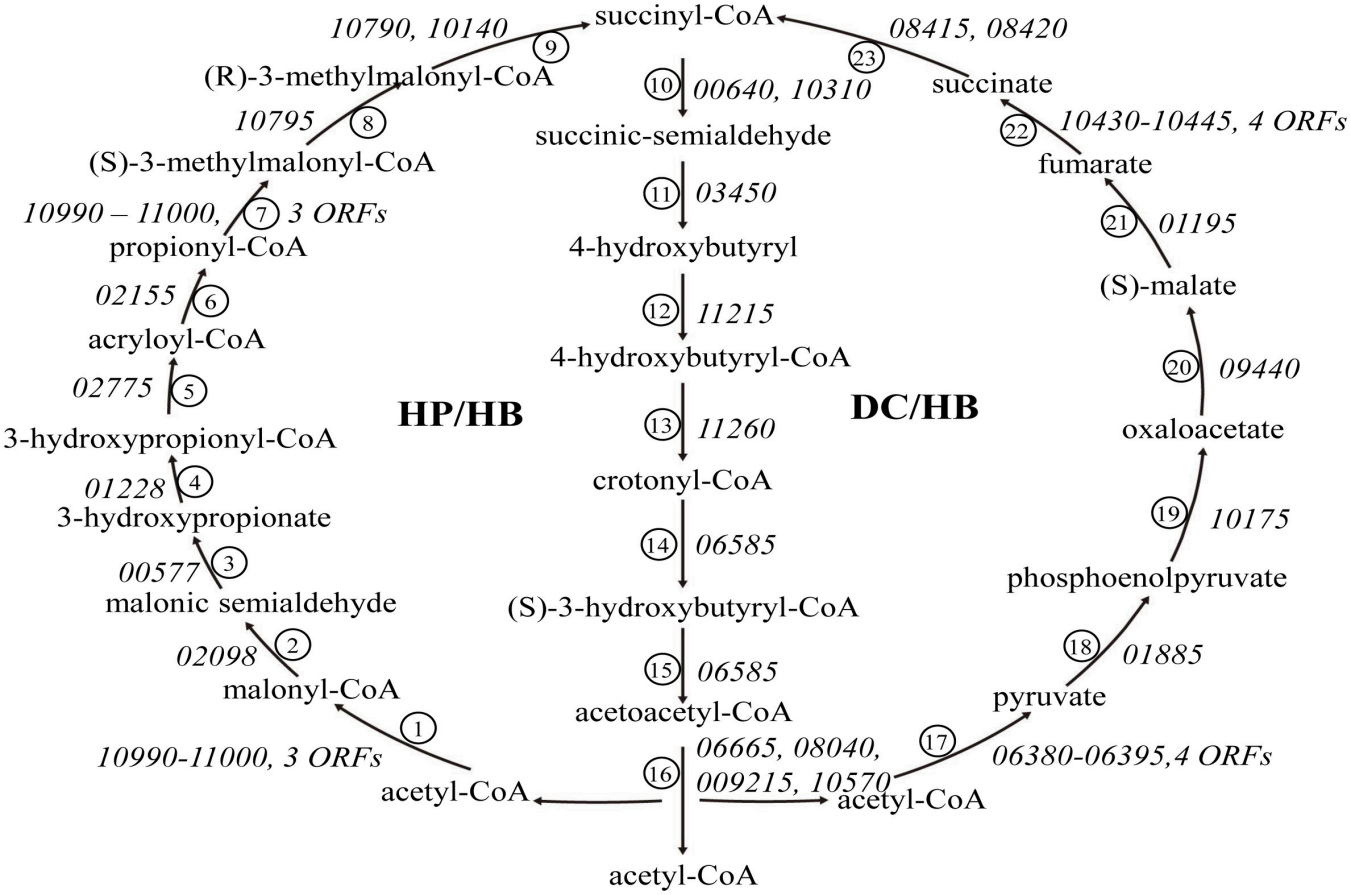
**The 3-hydroxypropionate/4-hydroxybutyrate (HP/HB) cycle and the dicarboxylate/ 4-hydroxybutyrate (DC/HB) cycle in ***Sulfolobus*****. Homologs of the enzymes in the two pathways in strain A20 are indicated by ORF numbers for the strain: ① acetyl-CoA caroxylase, ② malonyl-CoA reductase (NADPH), ③ malonate semialdehyde reductase (NADPH), ④ 3-hydroxypropionate-CoA ligase (AMP-forming), ⑤ 3-hydroxypropionyl-CoA dehydratase, ⑥ acrryloyl-CoA reductase (NADPH), ⑦ propionyl-CoA carboxylase, ⑧ methylmalonyl-CoA epimerase, ⑨ methylmalonyl-CoA mutase, ⑩ succinyl-CoA reductase, ⑪ succinic semialdehyde reductse (NADPH), ⑫ 4-hydroxybutyrate-CoA ligase (AMP forming), ⑬ 4-hydroxybutyryl-CoA dehydratase, ⑭ crotonyl-CoA hydratase, ⑮ (S)-3-hydroxybutyryl-CoA dehydrogenase (NAD^+^), ⑯ acetoacetyl-CoA beta-ketothiolase, ⑰ pyruvate synthase, ⑱ pyruvate:water dikinase, ⑲ PEP carboxylase, ⑳ malate dehydrogenase (NAD), ㉑ fumarate hydratase, ㉒ fumarate reductase, ㉓ succinyl-CoA synthetase (ADP-forming).

All tested *Sulfolobus* strains are mixotrophs capable of growing chemolithotrophically on CO_2_ with inorganic sulfur compounds (RISCs) as an energy source or heterotrophically on organic compounds (Brock et al., 1972; Keeling et al., 1998; Jan et al., 1999). Two CO_2_ fixation pathways, i.e., the 3-hydroxypropionate/4-hydroxybutyrate (HP/HB) cycle and the dicarboxylate/4-hydroxybutyrate (DC/HB) cycle, have been reported to exist in (hyper)thermophilic autotrophic Crenarchaeota (Berg et al., 2010). Like the other 17 *Sulfolobus* genomes, the strain A20 genome contains all of the genes encoding homologs of the enzymes of the two cycles (Figure 4).

#### Nitrogen metabolism

Like all other *Sulfolobus* genomes, the A20 genome contains genes encoding putative glutamate dehydrogenase (BFU36_RS08195), glutamine synthetase (BFU36_RS04000, BFU36_RS09525, and BFU36_RS10890) and the two subunits of carbamoylphosphate synthase (BFU36_RS02825 and BFU36_RS02830) (Table 4). It seems that all *Sulfolobus* strains employ a common strategy in the utilization of ammonium as a universal nitrogen source for the synthesis of glutamate, glutamine and carbamoyl-phosphate.

**Table 4:**
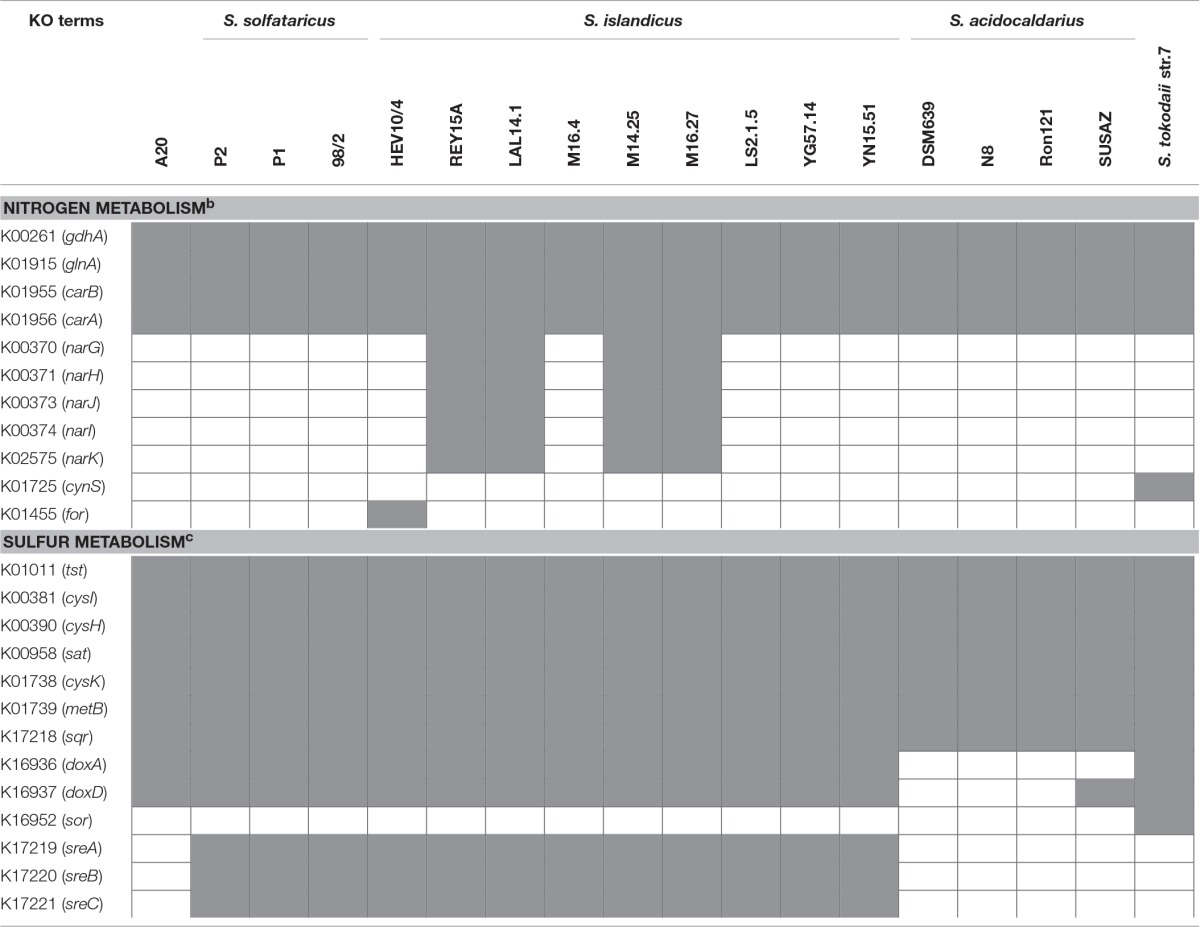
**Patterns of the distribution of genes encoding putative enzymes in nitrogen and sulfur metabolism in various ***Sulfolobus*** strains^a^**.

*^a^The presence of KO terms in nitrogen and sulfur metabolism is shown in gray*.

*^b^Nitrogen metabolism: gdhA, glutamate dehydrogenase (NAD(P)^+^) [EC 1.4.1.3]; glnA, glutamine synthetase [EC 6.3.1.2]; carB, carbamoyl-phosphate synthase large subunit [EC 6.3.5.5]; carA, carbamoyl-phosphate synthase small subunit [EC 6.3.5.5]; narG, nitrate reductase/nitrite oxidoreductase, alpha subunit [EC 1.7.5.1; 1.7.99.4]; narH, nitrate reductase/nitrite oxidoreductase, beta subunit [EC 1.7.5.1; 1.7.99.4]; narJ, nitrate reductase delta subunit; narI, nitrate reductase gamma subunit [EC 1.7.5.1; 1.7.99.4]; narK, nitrate/nitrite transporter; cynS, cyanate lyase [EC 4.2.1.104]; for, formamidase [EC 3.5.1.49]*.

*^c^Sulfur metabolism: tst, thiosulfate sulfurtransferase [EC 2.8.1.1]; cysI, sulfite reductase (NADPH) hemoprotein [EC 1.8.1.2]; cysH, phosphoadenosine phosphosulfate reductase [EC 1.8.4.8; 1.8.4.10]; sat, sulfate adenylyltransferase [EC 2.7.7.4]; cysK, cysteine synthase A [EC 2.5.1.47]; metB, cystathionine gamma-synthase [EC 2.5.1.48]; sqr, sulfide:quinone oxidoreductase [EC 1.8.5.4]; doxA, thiosulfate dehydrogenase [quinone] small subunit [EC 1.8.5.2]; doxD, thiosulfate dehydrogenase [quinone] large subunit [EC 1.8.5.2]; sor, sulfur oxygenase/reductase [EC 1.13.11.55]; sreA, sulfur reductase molybdopterin subunit; sreB, sulfur reductase FeS subunit; sreC, sulfur reductase membrane anchor*.

It is worth noting that four of the *S. islandicus* strains (i.e., REY15A, LAL14/1, M14.25, and M16.27) isolated from Iceland and Russia carry the *narGHJI* operon encoding a nitrate reductase and a nitrate transporter (*narK*) (Table 4), and, therefore, are potentially capable of utilizing nitrate. An operon encoding the subunits of urease (UreAB and UreC) and its accessory proteins (UreE, UreF, and UreG) is found in the genomes of *S. islandicus* HEV10/4, *S. tokodaii* str.7 and *S. metallicus* DSM 6482, suggesting that these strains are probably able to hydrolyze urea. Besides, genes for a putative cyanate lyase and a formamidase are found in the genomes of *S. tokodaii* str.7 and *S. islandicus* HEV10/4, respectively, suggesting a broader spectrum of nitrogen sources for these *Sulfolobus* strains.

#### Sulfur metabolism

All sequenced *Sulfolobus* genomes contain a gene cluster (BFU36_RS07995–BFU36_RS08005 in strain A20) coding for sulfite reductase, phosphoadenosine phosphosulfate reductase, and sulfate adenylyltransferase (Tables 4, 5). These enzymes probably catalyze the conversion of hydrogen sulfide into sulfite, and the subsequent transformation of sulfite into sulfate, with concomitant generation of ATP through substrate level phosphorylation (Kappler and Dahl, 2001; Rohwerder and Sand, 2007). A sulfide:quinine oxidoreductase (SQR) gene also exists in all *Sulfolobus* genomes (BFU36_RS09190 in strain A20). SQR may catalyze the oxidation of hydrogen sulfide into polysulfide (Rohwerder and Sand, 2007; Brito et al., 2009). Intriguingly, no homologs of sulfur oxygenase/reductase (SOR), a key enzyme for archaeal sulfur oxidation (Kletzin, 1992; Urich et al., 2006), are found in the genomes of *Sulfolobus* except for that of *S. tokodaii* str.7 (Kawarabayasi et al., 2001). The mechanism of elemental sulfur oxidization in *Sulfolobus* strains lacking SOR remains unknown. Putative genes for sulfur reductase (SRE) and thiosulfate:quinine oxidoreductase (TQO), which serve key roles in the reduction of elemental sulfur into hydrogen sulfide and the transformation of thiosulfate into tetrathionate, respectively (Laska et al., 2003; Guiral et al., 2005; Liu et al., 2012), are also found variably in *Sulfolobus* genomes (Tables 4, 5). Strain A20 and *S. tokodaii* str.7 carry *doxDA* (BFU36_RS07850–BFU36_RS07855), which encode a TQO homolog. *S. islandicus* and *S. solfataricus* have an SRE-encoding gene cluster (*sreABC*) and *doxDA*. *S. acidocaldarius* contains neither of the genes.

**Table 5:** **Predicted reactions in sulfur metabolism in ***Sulfolobus***^a^**.

**Strains**	**A20**	***S. acidocaldarius***	***S. solfataricus***	***S. islandicus***	***S. tokodaii***
**thiosulfate** + cyanide ⇋**sulfite** + thiocyanate (*tst*)	+	+	+	+	+
**hydrogen sulfide** + 3NADP^+^ + 3H_2_O ⇋**sulfite** + 3NADPH + 3H^+^ (*sqr*)	+	+	+	+	+
PAP + **sulfite** + thioredoxin disulfide ⇋**PAPS** + thioredoxin (*cysH*)	+	+	+	+	+
**APS** + diphosphate ⇋**sulfate** + ATP (*sat*)	+	+	+	+	+
**Sulfur**→**hydrogen sulfide** + **thiosulfate** (*sor*)	−	−	−	−	+
**Thiosulfate** + 6-decylubiquinone ⇋**tetrathionate** + 6-decylubiquinol (*doxAD*)	+	−	+	+	+
**Sulfur** + hydrogen ⇋**hydrogen sulfide** (*sre*)	−	−	+	+	−

a *APS, adenylyl sulfate; PAPS, 3′-phosphoadenylyl sulfate; PAP, adenosine 3′, 5′-bisphosphate*.

## Discussion

*Sulfolobus* sp. A20 was isolated from a hot spring in Costa Rica and the genomic DNA of the strain was completely sequenced. The addition of strain A20 to the growing list of the members of the genus *Sulfolobus* would aid further biogeographic comparison and evolutionary studies of this interesting group of archaea.

Sequence analysis indicates that strain A20 might be a mixotroph. The strain appears to be able to fix CO_2_ via the HP/HB cycle. It is also capable of metabolizing glucose through a branched-ED pathway and the TCA cycle, as are other *Sulfolobus* strains. In general, genes involved in central carbon metabolism are conserved in all sequenced *Sulfolobus* genomes. Some of the genes may exist in different numbers of copies and/or be arranged differently among different species, and the differences are in apparent agreement with the phylogenetic relationship rather than the geographical separation of the species (Figure 2). It is of interest that genes encoding enzymes for CO_2_ fixation through both HP/HB and DC/HB cycles are found in strain A20 and other sequenced *Sulfolobus* genomes. A similar finding has been reported for the genome of *Acidianus hospitalis* W1, a facultative anaerobe of the *Sulfolobales* (You et al., 2014). The two pathways differ in their sensitivity to oxygen, although they share many enzymes and intermediates in common (Ramos-Vera et al., 2011). The HP/HB cycle is more oxygen-tolerant than the DC/HB cycle since pyruvate synthase, one of key enzymes in the latter cycle, is oxygen sensitive (Jahn et al., 2007; Huber et al., 2008). As aerobes or microaerobes, members of the Sulfolobales have been shown to fix CO_2_ through the HP/HB cycle. However, genes coding for putative pyruvate synthase, pyruvate:water dikinase and PEP carboxylase in the DC/HB cycle were found to be expressed, although at a low level, in *Metallosphaera sedula*, an aerobe closely related to *Sulfolobus* strains (Berg et al., 2010). Therefore, we infer that the DC/HB pathway may also be employed by *Sulfolobus* to fix CO_2_ under certain conditions.

Similarly, genes involved in the two ED pathways, i.e., the semi-phosphorylated pathway and the non-phosphorylated pathway, are also conserved in all the sequenced *Sulfolobus* genomes. The two ED pathways were named as the archaeal branched ED pathway (Sato and Atomi, 2011), and their functions were verified in *S. solfataricus* (Ahmed et al., 2005). The redundancy of the pathways for central carbon metabolism in *Sulfolobus* may contribute to the adaption of the organisms to thriving in the extreme and oligotrophic habitats.

All *Sulfolobus* genomes contained a complete pathway for ammonium assimilation, which is similar to that found in heterotrophic bacteria (Zalkin, 1993; Guo et al., 2011; Wang et al., 2016), suggesting that *Sulfolobus* prefers to use ammonia as the nitrogen source. Strain A20 is probably unable to use other inorganic nitrogen sources for growth, while several of the *S. islandicus* strains and *S. tokodaii* str.7 might be able to use nitrate, urea, cyanate or formamide as their nitrogen source. These results point to the diversity of nitrogen utilization by *Sulfolobus*. It remains to be determined if the difference in the ability of *Sulfolobus* strains to use inorganic nitrogen compounds correlates with the availability of the nitrogen sources in the habitats of the strains.

Genomic analyses reveal the presence of transposase genes and repeating sequences near the *nar* gene cluster, suggesting the potential mobility of the cluster. The *nar* cluster was found at either of the two genomic sites in four *S. islandicus* strains containing the cluster. In the two *S. islandicus* strains from Iceland (i.e., REY15A and LAL14/1), the *nar* cluster resides on the complementary strand downstream of a sequence encoding a GntR family transcriptional regulator, a CoA ester lyase and an esterase (SIRE_RS02235–SIRE_RS02245 in REY15A and SIL_RS02325–SIL_RS02335 in LAL14/1). This site of potential *nar* insertion is termed insertion site A. On the other hand, in the two strains from Kamchatka (i.e., M16.27 and M14.25), the cluster is located downstream of a sequence encoding a 3-hydroxyacyl-CoA dehydrogenase, an AMP-dependence synthetase and an acetyl-CoA synthetase (M1627_RS04095–M1627_RS04105 in M16.27 and M1425_RS04080–M1425_RS04090 in M14.25). We denote this potential location for the insertion of the *nar* cluster insertion site B. Although only two strains were found to contain the *nar* cluster at insertion site A, this insertion site is present in all *Sulfolobus* strains analyzed in this study. Variation occurs downstream of the site. There are seven types of gene organization downstream of insertion site A in the 18 strains (Tables S3, S4). The tandem array of the three genes at insertion site B is found only in *S. islandicus* strains isolated form Kamchatka, Yellowstone National Park (YNP), and Lassen in USA (Tables S3, S4). Three general patterns of gene arrangement were identified at insertion site B. The two *S. islandicus* strains from USA (i.e., L.S 2.15 and Y57.14) are of one type, and the two Kamchatka *S. islandicus* strains (i.e., M16.27 and M14.25) belong to the other type. Remarkable variation in gene arrangement indicates that the two sites are where active transposition has taken place. The biogeographical difference in genomic location of the *nar* gene cluster presumably resulted from the transposition of the cluster. Since the presence of the *nar* cluster is restricted to *S. islandicus* and some of the strains in this species lack the gene cluster, we hypothesize that the species originally carried the cluster. When it spread to various geographical locations, loss or transposition of the gene cluster occurred, producing variants that thrive in various parts of the globe today. Whether the *nar* cluster was originally acquired through horizontal gene transfer is unclear. However, no significant difference in GC content between the gene cluster and the genome was detected.

Elemental sulfur metabolism is complex in *Sulfolobus*, and relatively low conservation in sulfur metabolism exists among the sequenced genomes. Strain A20 is likely capable of utilizing hydrogen sulfide because of the presence in its genome a conserved gene cluster for sulfur metabolism (Kawarabayasi et al., 2001; Chen et al., 2005). Although most *Sulfolobus* strains have been described as sulfur-oxidizing microbes (Brock et al., 1972), the biochemical process of elemental sulfur oxidation has yet to be fully understood. The *sor* gene encoding the classical sulfur oxygenase/reductase required for the initial step in the archaeal sulfur oxidation pathway (Urich et al., 2006) is present in none of the sequenced *Sulfolobus* genomes except for the genome of *S. tokodaii* str.7 (Kawarabayasi et al., 2001; She et al., 2001; Chen et al., 2005; Guo et al., 2011; Jaubert et al., 2013). Instead, there is a gene cluster encoding sulfur reductase (SRE), which reduces S^0^ with the help of a hydrogenase in anaerobically grown *Acidianus ambivalens* (Laska et al., 2003), in the genomes of *S. solfataricus* and *S. islandicus*. However, no hydrogenase genes have been identified in the two species. So, whether and how the sulfur reductase catalyzes sulfur reduction in the absence of a hydrogenase under aerobic conditions remains to be determined. It has been reported that *Sulfolobus tokodaii* str.7 grows poorly in the presence of elemental sulfur under the facultatively chemolithotrophic conditions (Suzuki et al., 2002), although it encodes a homolog of the classical sulfur oxygenase/reductase. However, the strain was able to oxidize hydrogen sulfide into sulfate (Kawarabayasi et al., 2001), suggesting the possibility of functional divergence of the homologs of sulfur oxygenase/reductase in *Sulfolobus*. Therefore, further investigation is needed to understand the mechanisms underlining elemental sulfur metabolism in *Sulfolobus*.

The *sre* gene cluster is flanked upstream by a hypothetical protein and a 4Fe-4S ferredoxin and downstream by another 4Fe-4S ferredoxin and two hypothetical proteins (Tables S3, S4). This entire sequence is located downstream of a *cupin* gene. Based on the presence of genes between *cupin* and the *sre* cluster, three types of gene arrangement were identified at this site. A transposase gene is located between *cupin* and the *sre* cluster in *S. solfataricus* strains P1 and P2, both of which were isolated from Naples, Italy. However, no transposase gene at this site was found in *S. solfataricus* strain 98/2 or *S. islandicus* strains from YNP. Instead, a gene for the large subunit of nitricoxide redutase is present at this site in these strains. By comparison, a pseudogene is in the place of the transposase gene in *S. islandicus* strain 14.25 from Kamchatka. The two other *S. islandicus* strains (i.e., M16.4 and M16.27) from Kamchatka contain multiple transposase genes as well as hypothetical proteins at the site. Patterns of gene arrangement upstream of the *sre* gene cluster appear to carry distinct geographical markers, since they exhibit similarity among closely located strains of the same species. Whether the function of the *sre* gene cluster is affected by its genomic environment is unclear.

A putative *tusA-dsrE2-dsrE3A* gene cluster is linked to the *hdr* cluster (*hdrC1*-*hdrB1A*-*hyp*-*hdrC2*-*hdrB2*) in all *Sulfolobus* genomes. The *hdr* cluster encodes a heterodisulfide-reductase complex, which may be involved in sulfur transfer and reversible reduction of the disulfide bond X-S-S-X in *Acidithiobacillus ferrrooxidans* (Quatrini et al., 2009; Liu et al., 2014), while the *tusA-dsrE2-dsrE3A* gene cluster may encode functions in the transformation of tetrathionate into thiosulfate in *Metallosphaera cuprina* (Liu et al., 2014). How the two genomically linked gene clusters function in sulfur metabolism remains to be understood.

Taken together, our genomic analyses reveal that these *Sulfolobus* species are conserved in central carbon metabolism, but differ in the ability to use inorganic nitrogen and sulfur sources. The ability of *Sulfolobus* to utilize nitrate or sulfur is encoded by a gene cluster flanked by IS elements or their remnants. These clusters appear to have become fixed at a specific genomic site in some strains and lost in other strains during the course of evolution.

## Author contributions

XD and LH designed the project. XD and ZZ analyzed the data. HW, LW, YZ, ZD, MM-L, and WH-A collected sample, purified the strain and prepared the genomic DNA for sequencing. KL and XZ performed bioinformatic analysis of the genome sequences. CJ and CL analyzed the pathways of sulfur metabolism. LH, XD and ZZ wrote the manuscript.

### Conflict of interest statement

The authors declare that the research was conducted in the absence of any commercial or financial relationships that could be construed as a potential conflict of interest.
