# Supplementary material for: Genome Sequencing of Sulfolobus sp. A20 from Costa Rica and Comparative Analyses of the Putative Pathways of Carbon, Nitrogen, and Sulfur Metabolism in Various Sulfolobus Strains
Source: Front Microbiol. 2016 Nov 30;7:1902. doi: 10.3389/fmicb.2016.01902 (PMC5127849; doi:10.3389/fmicb.2016.01902)
Supplement: Supplementary file 2 [file Presentation1.PDF]

Supplementary materials

Table S1. Core genes of the genus *Sulfolobus* in strain A20. See the excel file in appendix.

Table S2. Strain A20 genes encoding enzymes and proteins involved in carbon metabolic pathways discussed in the manuscript.

gene locus_tag	Annotation	Blast to the database of KEGG (KO term)
BFU36_RS00560	trehalose/maltose import ATP-binding protein MalK	(K17314):treV trehalose transport system ATP-binding protein
BFU36_RS00565	trehalose/maltose transport system permease protein MalG	(K17313):treU trehalose transport system permease protein
BFU36_RS00570	trehalose/maltose transport system permease protein MalF	(K17312):treT trehalose transport system permease protein
BFU36_RS00575	trehalose/maltose-binding protein MalE precursor	(K17311):treS trehalose transport system substrate-binding protein
BFU36_RS00855	maltotriose-binding protein precursor	(K15770):cycB arabinogalactan oligomer / maltooligosaccharide transport system substrate-binding protein
BFU36_RS00860	trehalose/maltose transport system permease protein MalF	(K15771):ganP arabinogalactan oligomer / maltooligosaccharide transport system permease protein
BFU36_RS00865	trehalose/maltose transport system permease protein MalG	(K15772):ganQ arabinogalactan oligomer / maltooligosaccharide transport system permease protein
BFU36_RS00870	trehalose/maltose import ATP-binding protein MalK	(K10112):msmX multiple sugar transport system ATP-binding protein
BFU36_RS07010	lycopene cyclase	-
BFU36_RS07015	phytoene synthase	(K02291):crtB phytoene synthase [EC:2.5.1.32]
BFU36_RS07020	porin	(K15746):crtZ beta-carotene 3-hydroxylase [EC:1.14.13.129]
BFU36_RS07025	phytoene desaturase	(K10027):crtI phytoene desaturase [EC:1.3.99.26 1.3.99.28 1.3.99.29 1.3.99.31]
BFU36_RS07440	sugar ABC transporter substrate-binding protein	(K10196):ABC.GLC.S glucose/arabinose transport system substrate-binding protein
BFU36_RS07445	trehalose/maltose transport system permease protein MalF	(K10197):ABC.GLC.P glucose/arabinose transport system permease protein
BFU36_RS07450	trehalose/maltose transport system permease protein MalG	(K10198):ABC.GLC.P1 glucose/arabinose transport system permease protein
BFU36_RS07455	trehalose/maltose import ATP-binding protein MalK	(K10199):ABC.GLC.A glucose/arabinose transport system ATP-binding protein
BFU36_RS08120	trehalose/maltose import ATP-binding protein MalK	(K10199):ABC.GLC.A glucose/arabinose transport system ATP-binding protein
BFU36_RS08125	trehalose/maltose transport system permease protein MalG	(K10198):ABC.GLC.P1 glucose/arabinose transport system permease protein
BFU36_RS08130	trehalose/maltose transport system permease protein MalF	(K10197):ABC.GLC.P glucose/arabinose transport system permease protein

BFU36_RS09315	trehalose synthase	(K13057):treT trehalose synthase [EC:2.4.1.245]

9

Table S3. Distribution of the insertion sites for the *nar* and *sre* gene clusters and the variants of the sites[1].

Species	Strain	Iceland [2]	Kamchatka	YNP/ Lassen	Naples	Ronneburg	Japan	Los Azufres	Costa Rica
S. islandicus	REY15A	A-1<br>−[3]B<br>− S							
	LAL14/1	A-I<br>− B<br>− S							
	HVE10/4	A-I<br>− B<br>− S							
	M14.25		A-II<br>B-I<br>S-III						
	M16.27		A-II<br>B-I<br>S-III						
	M16.4		A-II<br>B-II<br>S-III						
	Y57.14			A-III<br>B-II<br>S-II					
	YN15.51			A-III<br>B-III<br>S-II					
	L.S.2.15			A-I<br>B-III<br>S-II					
S. solfat aricu	98/2			A-IV<br>− B<br>S-II					

	P2				A-IV<br>− B<br>S-I				
	P1				A-IV<br>− B<br>S-I				
S. acidocaldarius	DSM639		A-VI<br>− B<br>− S						
	N8						A-VI<br>− B<br>− S		
	Ron121					A-VI<br>− B<br>− S			
	SUSAZ							A-VI<br>− B<br>− S	
S. tokodaii	str.7						A-VII<br>− B<br>− S		
	Strain A20								A-V<br>− B<br>− S

[1] See Table S4 for the designations of the insertion sites.

[2] Sites of strain isolation: see also Table 1.

[3] The absence of an insertion site is indicated by ‘-’.

Table S4. Sites where the *nar* and the *sre* gene clusters are inserted[1].

Strain	Subtype[2]	Genes at insertion site and its variants
		Insertion site A for *nar*
S. islandicus HVE10/4 (Hvergaardi, Iceland) L.S.2.15 (Lassen, USA)	A-I	**GntR family transcriptional regulator – CoA ester lyase – esterase**[3] **–** ATPase (pseudo) – X (11-57)[4] – *IS630 family transposase*[5]
S. islandicus REY15A and LAL14/1 (Reykjanes, Iceland)	A-I (*nar*)	**GntR family transcriptional regulator – CoA ester lyase – esterase –** ATPase (pseudo) – X (2-4) – *narI – narJ – narH – narG – narK* – X (1-4) – IS630 family transposase
S. islandicus M14.25, M16.27 and M16.4 (Kamchatka, Russia)	A-II	**GntR family transcriptional regulator – CoA ester lyase – esterase** – ATPase – X (21-26) – *IS110 family transposase*
S. islandicus Y57.14 and YN15.51 (YNP, USA)	A-III	**GntR family transcriptional regulator – CoA ester lyase – esterase** – transposase (pseudo) **-** *transposase*
S. solfataricus P2 and P1 (Naples, Italy) strain 98/2 (YNP, USA)	A-IV	**GntR family transcriptional regulator – CoA ester lyase – esterase** – DNA binding protein – DNA polymerase subunit beta – *transposase (pseudo) – IS4 family transposase*
Sulfolobus sp. A20 (Las Palias, Costa Rica)	A-V	**GntR family transcriptional regulator – CoA ester lyase – esterase** – hy[6] – hy – FAD-linked oxidase – phosphoenolpyruvate carboxykinase ……
S. acidocaldarius DSM639 (YNP, USA*)* N8 (Hokkaido, Japan) Ron121 (Ronneburg, Germany)	A-VI	ligase – hy – **CoA ester lyase – esterase** – hy – electron transfer flavoprotein subunit alpha – FAD-dependent oxidoreductase – ferredoxin family protein ...
S. tokodaii str.7 *(*Kyushu, Japan*)*	A-VII	*peroxiredoxin* – **CoA ester lyase** – 2-ketoisovalerate ferredoxin oxidoreductase – pyruvate ferredoxin oxidoreductase – ferredoxin pyruvate synthase …
		Insertion site B for *nar*
S. islandicus M14.25 and M16.27 (Kamchatka, Russia)	B-1	**3-hydroxyacyl-CoA dehydrogenase – AMP-dependent synthetase – acety-CoA synthetase** – hy – *narK – narG – narH – narJ – narI* – hy – hy – hy – *transposase*
S. islandicus M16.4 (Kamchatka, Russia) Y57.14 (YNP, USA)	B-II	**3-hydroxyacyl-CoA dehydrogenase – AMP-dependent synthetase – acety-CoA synthetase** – X(1-4) – *transposase*
S. islandicus YN15.51 (YNP, USA) *S. islandicus* L.S.2.15 (Lassen, USA)	B-III	**3-hydroxyacyl-CoA dehydrogenase – AMP-dependent synthetase – acety-CoA synthetase** – pyridine nucleotide-disulfide oxidoreductase – acetoin:2,6-dichlorophenolindophenol oxidoreductase subunit alpha – TPP-dependent aceoin dehydrogenase complex E1 protein subunit beta – ATP-NAD kinase …
		Insertion site for *sre*
S. solfataricus P1 and P2	S-I	**Hy – cupin** –hy (pseudo) – *transposase* – hy – hy –

(Naples, Italy)		4Fe-4S ferredoxin – *sreC – sreB – sreA* – hy – 4Fe-4S ferredoxin
S. islandicus Y57.14 and YN15.51 (YNP, USA)<br>*S. islandicus* L.S.2.15 (Lassen, USA)<br>*S. solfataricus* 98/2 (YNP, USA)	S-II	**Hy – cupin** – nitric oxide reductase large subunit – hy – 4Fe-4S ferredoxin – *sreC – sreB – sreA* – 4Fe-4S ferredoxin – X (4-9) – *transposase*
S. islandicus M16.27 and M16.4 (Kamchatka, Russia)	S-III	**Hy – cupin** – *transposase* – *transposase* – X(4-7) – ***transposase*** – hy – 4Fe-4S ferredoxin – *sreC – sreB – sreA* – 4Fe-4S ferredoxin – X(4) – *transposase*
S. islandicus M14.25 (Kamchatka, Russia)	S-III*	**Hy – cupin** – *nitric oxide reductase (pseudo)* – hy -4Fe-4S ferredoxin – *sreC – sreB – sreA* – 4Fe-4S ferredoxin — X(4) – *transposase*

[1] The genomic sites for the insertion of the *nar* and *sre* gene clusters and variants of these sites are listed. A 5’-to-3’ linear array of genes at each site is given, but the orientation of each gene is not shown.

[2] Each insertion site is classified into subtypes based on sequence variation.

[3] A conserved stretch of genes characteristic of an insertion site is shown in bold face.[4] Variable genes are represented by ‘X’, and the number of these genes is shown by a number or a range in parentheses.

[5] Genes encoding a transposase are indicated in italics.

[6] Genes encoding a hypothetical protein are indicated by ‘hy’.
